# Different Performance of *Phthorimaea operculella* Zeller (Lepidoptera: Gelechiidae) among Four Potato Tuber Varieties under Laboratory Condition

**DOI:** 10.3390/insects12070580

**Published:** 2021-06-25

**Authors:** Mengdi Zhang, Junjie Yan, Abid Ali, Yulin Gao

**Affiliations:** 1 State Key Laboratory for Biology of Plant Diseases and Insect Pests, Institute of Plant Protection, Chinese Academy of Agricultural Sciences, Beijing 100193, China; sdaumengdizhang@163.com (M.Z.); junjieyan0125@163.com (J.Y.); 2 College of Agronomy, Inner Mongolia Agricultural University, Hohhot 010019, China; 3 Department of Entomology, Faculty of Agriculture, University of Agriculture, Faisalabad 38040, Pakistan; abid_ento74@yahoo.com; 4 College of Life Science, Shenyang Normal University, Shenyang 110034, China; 5 National Center of Excellence for Tuber and Root Crop Research, Chinese Academy of Agricultural Sciences, Beijing 100081, China

**Keywords:** potato tuber moth, *Phthorimaea operculella*, potatovarieties, performance behavior, agricultural control

## Abstract

**Simple Summary:**

*Phthorimaea operculella* is considered one of the most damaging potato pests in both field and storage. Learning the developmental performance of *P. operculella* on different potato varieties is necessary before developing management practices.

**Abstract:**

The potato tuber moth *Phthorimaea operculella* Zeller is one of the most damaging potato pests in the world and is difficult to control as the larvae feed only inside potatoes. Evaluating the effects of performance of *P. operculella* on different potato varieties may help to facilitate the development of effective pest management practices. In our study, *P. operculella* reared on potato variety Lishu6 showed strong performance and on Qingshu 9 exhibited the poorest performance, while *P. operculella* fed on Hezuo 88 and Yunshu 304 performed moderately. Firstly, larval performance of *P. operculella* fed on Lishu 6 with that on Hezuo 88, Yunshu 304, and Qingshu 9 was compared by using an age-stage two-sex life table. Compared with larvae fed on Lishu 6, those fed on Qingshu 9 exhibited significantly lower survival, with only 5.56% developing to the adult stage. Secondly, the pupal weight experiment showed that the pupae weight of *P. operculella* feeding on Lishu 6 tubers (0.0123 g) was significantly heavier than that those feeding on Qingshu 9 (0.0102 g) (*p* < 0.001). Thirdly, female oviposition and larvae feeding preference indicated that females oviposited on Lishu 6 in preference to Qingshu 9 (*p* < 0.05, *p* < 0.001). Overall, this study demonstrated that Qingshu 9 is the least suitable host, and planting this variety over a large scale can provide a basis for the next step of *P. operculella* control.

## 1. Introduction

The potato tuber moth, *Phthorimaea operculella* Zeller (Lepidoptera: Gelechiidae), is a cosmopolitan pest, distributed among tropical and subtropical countries in South, Central, and North America, Oceania, Africa, Australia, Europe, and Asia [1,2], restricted largely to solanaceous crops especially potatoes (*Solanum tuberosum* L.) [3]. Larvae of this species mine leaves, stems, and petioles of potato plants, and excavate tunnels throughout potato tubers. However, typical damage results from larvae mining the tubers [4].

Potato (*S. tuberosum* L.) is the fourth major food crop around the world after rice (*Oryza sativa* L.), wheat (*Triticum* spp. L.), and maize (*Zea mays* L.) [5,6], and economically one of the important leading species in Southwest China (e.g., especially in Yunnan province) [7]. With the continuous expansion of potato planting area and its vigorous industrial development, potato plays a vital role in ensuring national food security, targeted poverty alleviation and structural adjustment of planting industry in China [6]. However, potato production is challenged by many factors including pests and diseases, particularly *P. operculella*. Foliar damage by *P. operculella* to the potato plants does not usually cause significant yield losses [8]. However, infested tubers may reduce marketability, and damage can be distinctive in storage, especially in non-refrigerated systems [9]. Moreover, *P. operculella* is responsible for about 20–30% infestation in the field, and 100% under storage [10]. As most of the economic damage occurs when *P. operculella* infests the tubers, so researchers should focus on management of this insect pest during tuber period. 

Finding effective methods to control *P. operculella* has become more and more important for the healthy development of the potato industry in the world. Moreover, increasing damage is due to the infestation characteristics of *P. operculella* of burrowing, high reproductive capacity, multivoltinism, and adaptability to changing climates [11,12] because of frequently transported seed tubers. The early control and management measures are mainly focused on the natural tuber resistance [6]. Although it is difficult to achieve effective control by a single method when the infestation is very high, any individual method may be effective when populations are low [6]. According to previous studies, there was different behavior performance of *P. operculella* among different tuber varieties [13]. Moreover, Mansouri et al. [14] reported different larval survival and developmental times among different varieties while comparing eight commercial potato cultivars and four Iranian tuber germplasms, indicating those performance of pests can be an important component for resistance to *P. operculella* and pest management. Therefore, a thorough knowledge of its biological performance of *P. operculella* is necessary before developing sustainable management practices.

Thus, most commonly planted four different potato varieties in Yunnan Province were selected to compare the biological performance of *P. operculella* under laboratory conditions. For this, the characteristics of life history of *P. operculella* larvae fed on four different tuber varieties were compared by using the age-stage two-sex life table method. Moreover, immature stages with respect to their feeding performance on various host plants and the subsequent adult performance were assessed.

## 2. Materials and Methods

### 2.1. Insects

The initial population of *P. operculella* was collected during June 2013 from potato (*S. tuberosum*) fields located in Kunming, Yunnan Province, China (E 103.79, N 25.51). An experimental population was established according to the method described by Gui [15]. The whole growth period of *P. operculella* was completed in Sanyo artificial environmental chamber (MLR-351H, SANYO Electric Co., Ltd., Moriguchi City, Osaka, Japan) under controlled conditions of 26 ± 1 °C, L:D = 12 h:12 h, and 70% ± 10% RH. The populations were reared for more than three generations to ensure homogeneity of *P. operculella* culture prior to their use in the experiments. After the collection of female laid egg masses, they were deposited in glass tubes. For further experimental use, all larval instars of *P. operculella* were collected. Both males and female adults were fed on 10% (*w*/*v*) mixture prepared with honey and water.

### 2.2. Potato Tubers

In this study, four tuber varieties including Lishu 6, Hezuo 88, Yunshu 304, and Qingshu 9 with the similar shape (ellipse), consistent weight, and the same number of eyebuds obtained from Yunnan Agricultural University, Yunnan Province, China, were selected for their fitness and preference studies.

### 2.3. Life History Characteristics of P. operculella Cultured on Four Potato Tuber Varieties

The development, survival, and reproduction of *P. operculella* fed on four different potato tuber varieties as mentioned above were investigated and compared. The newly hatched larvae total of 180 (on average 60 larvae per glass containers, n = 180) using a soft brush were transferred to three separate glass containers (25 cm diameter × 10 cm depth fitted with mesh lids) and placed in tuber (around 600 g each container) surface which was washed with running water and then drying on sterile paper [4]. The containers were kept in a climatic chamber described as above. After daily recording the survival and developmental time for each of larval and pupal stage pupae were placed individually into centrifugal tubes (2 mL). The newly emerged males and females were individually paired engaged from the colony in plastic chambers (height × diameter = 14 cm × 6 cm) covered with a fine mesh for ventilation and a mixture of 10% (*w*/*v*) honey in distilled water was provided to these pairs for their feeding. Filter paper (diameter = 5 cm) around the plastic chambers was used as the oviposition substrate and was replaced on daily basis. Every day until all females died, the number of individual female eggs masses laid were recorded. The survivorship, fecundity, oviposition period, and female longevity were noted. Newly hatched each larva was counted as single replication [16].

### 2.4. Pupal Weight of P. operculella on Four Different Potato Tuber Varieties

To examine the effects of different tuber varieties on pupal weight of *P. operculella*, 180 newly hatched larvae were transferred to four different potato tuber varieties following the same procedures described above. Once the *P. operculella* reached pupal stage, 40 pupae were harvested and randomly weighted using Electro balance (Mettler Toledo, Shanghai, China). Each replication contained 40 pupae, the whole bioassays were repeated five times using independent batches of pupae and plants.

### 2.5. Oviposition Preference of P. operculella Females on Four Different Potato Tuber Varieties

To determine the oviposition preference of *P. operculella* females among four different potato tuber varieties, choice and non-choice tests were conducted in four potted plants (positioned in a square). In the choice test, four different potato tuber varieties (around 150 g for each of Lishu 6, Hezuo 88, Yunshu 304, and Qingshu 9) were enclosed in a plastic cage (length × width × height = 25 cm × 25 cm × 25 cm), positioned in a square. In the non-choice test, one tuber of each variety (around 150 g) was separately enclosed in the center of a similar plastic cage (length × width × height = 20 cm × 20 cm × 20 cm). For both tests, five pairs (males and females) were allowed to mate for 3 days after their release into the cage, and after that, all moths and tubers were removed from the cage. The number of egg masses present on each tuber and the inner wall of the cages were recorded. Each caged plant was maintained in a room chamber under same conditions as explained above. Petri dishes with 10% (*w*/*v*) honey–water mixture were provided during the test. For each test, in total ten replications were carried out where each cage responded as a single replication in both choice test and no-choice test.

### 2.6. Feeding Preference of P. operculella Larvae on Four Different Potato Tuber Varieties

According to the method of Wang et al. [17], cut tubers (2 cm × 2 cm × 1 cm) of different varieties were placed in equidistance on a glass petri dish (diameter × height = 20 cm × 3.5 cm). Twenty-four newly hatched 1st, instar larvae were manually released on the middle of the petri dishes to freely choose the tubers, and then covered with perforated seal membrane for ventilation. The petri dishes were kept in a climatic chamber described as above. After 24 h, the number of larvae inside each tuber were observed by cutting tubers into slices. The experiment was repeated 10 times. All larval instars followed the same method as described above.

### 2.7. Data Analysis

Based on an age-stage two-sex life table [18], data of the life history of all *P. operculella* individuals were analyzed and constructed using the computer program TWO-SEX-MSChart [19]. The survival rate (*s_xj_*) (*x* = age, *j* = stage) and fecundity (*f_xj_*) were computed by following the method described by Chi and Liu [20]. The age-specific survival rate (*l_x_*) and the age-specific fecundity (*m_x_*) were then calculated as follows:

$$l_{x}=\sum_{j=1}^{k}s_{xj},$$

$$m_{x}=\frac{\sum_{j=1}^{k}s_{xj}f_{xj}}{\sum_{j=1}^{k}s_{xj}}.$$

The intrinsic rate of increase (*r*) was calculated according to the Euler–Lotka formula with the age indexed from 0 as follows [21]:

$$\sum_{x=0}^{\infty }e^{-r\left(x+1\right)}l_{x}m_{x}=1.$$

The finite rate (*λ*), the net reproductive rate (*R*_0_), and the mean generation time (*T*) were individually estimated as follows:

$$\lambda =e^{r},$$

$$R_{0}=\sum_{x=0}^{\infty }l_{x}m_{x},$$

$$T=\left(lnR_{0}\right)/r.$$

The variances and standard errors of the population parameters were measured by the bootstrap technique [22,23], while significantly different host plants were calculated with the paired bootstrap test.

To estimate the pupal weight and the choice test of *P. operculella* among four different potato tuber varieties, One-way analysis of variance (ANOVA) and Tukey’s HSD post-hoc test were used. In the non-choice test, *t*-test was used to compare the distribution of egg masses on plants and the cage walls. Differences were statistically considered significant at *p* < 0.05. All statistical analyses in the present study were conducted in the R software environment (v.4.0.3,) (R Development Core Team, 2014, Auckland, New Zealand).

## 3. Results

### 3.1. Development, Survivorship, and Reproduction of P. operculella on Four Different Potato Tuber Varieties

All developmental periods of each immature stage, female fecundity, and adult longevity of *P. operculella* fed on four tuber varieties are present by Table 1. The larval and pupal developmental stage was significantly lower on Qingshu 9 (larval: 15.50 d, pupa: 9.90 d) than on Lishu 6 (larval: 14.18 d, pupa: 8.69 d) (all *p-*values <0.001). Statistically significant longer adult longevity was accounted for male and female of *P. operculella* developing on Lishu 6 (female: 14.28 d, male: 18.03 d) than on Qingshu 9 (female: 9.40 d, *p* < 0.001; male: 12.80 d, *p* < 0.05). The mean fecundity of females fed on Lishu 6 was 213 eggs, which was significantly higher that of those on Hezuo 88 (147 eggs), Yunshu 304 (142 eggs), and Qingshu 9 (74 eggs) (all *p*-values <0.001).

**Figure 1:**
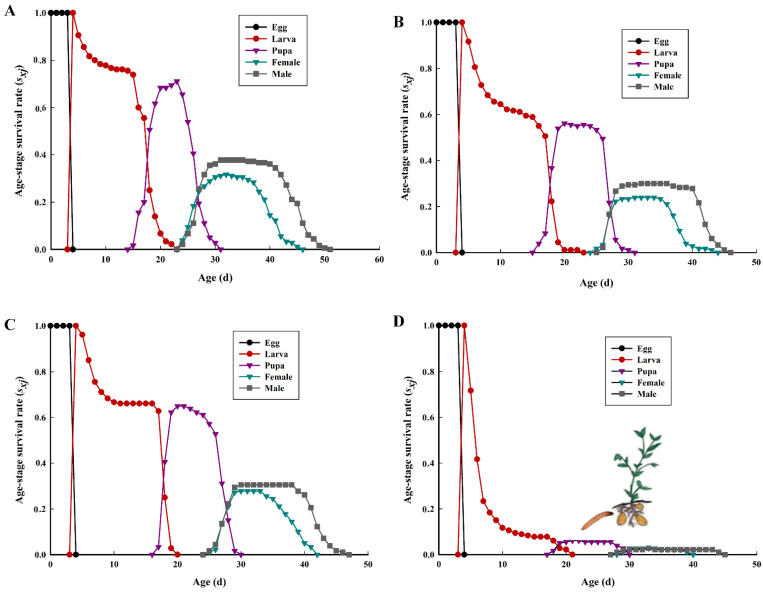
Age-stage specific survival rates (s*_xj_*) of *P. operculella* on four different potato tuber varieties. (**A**): Lishu 6; (**B**): Hezuo 88; (**C**): Yunshu 304; (**D**): Qingshu 9.

**Figure 2:**
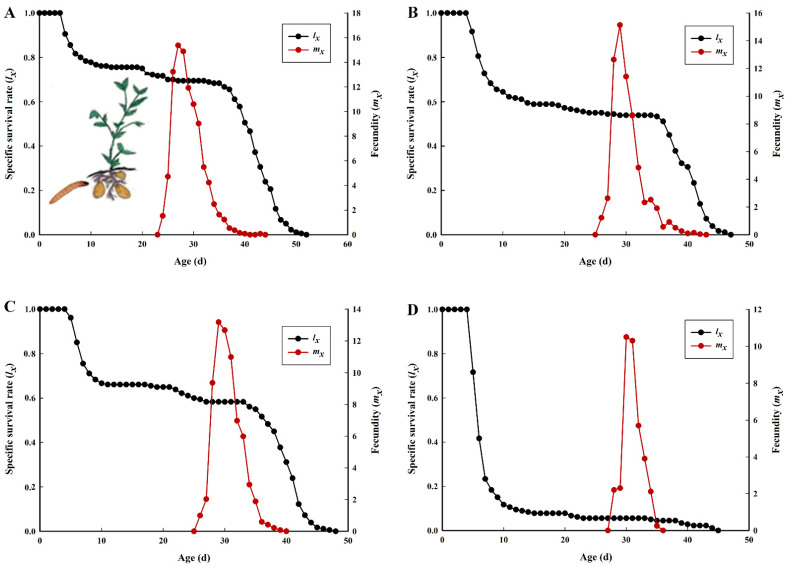
Age-specific survival rate (*l_x_*) and fecundity (*m_x_*) of *P. operculella* on four different potato tuber varieties. (**A**): Lishu 6; (**B**): Hezuo 88; (**C**): Yunshu 304; (**D**): Qingshu 9.

The probability depicted in the curves of the age-stage survival rate (*s_xj_*) indicates that a newborn will develop to stage *j* and survive to stage *x* (Figure 1). The overlapping stage-specific survivorship curves resulted due to variable developmental rates among individuals [24]. The probability difference among the four different potato tuber varieties accounting for 70% on Lishu 6, higher than on Qingshu 9 (5.56%), describes that a newly hatched neonate of *P. operculella* will survive to the adult stage. Higher mortality rates occurred in the larvae when fed on Qingshu 9 potato tubers, with mortality rates of nearly 92.22%. The age-specific survival rate (*l_x_*) and age-specific fecundity (*m_x_*) are plotted in Figure 2. The *lx* curve describes the change in the survival rate of the cohort with age and shows that *P. operculella* fed on Qingshu 9 had a quick survivorship decline starting around day 4. The *m_x_* curve shows that reproduction began at age 24 days and 28 days in *P. operculella* fed on Lishu 6 and Qingshu 9, respectively. For *P. operculella* fed on Lishu 6, the maximal daily fecundity was at age 27 days with mean fecundity of 15 eggs, higher than the corresponding value for *P. operculella* fed on Qingshu 9 (30 days, 10 eggs).

### 3.2. Population Parameters of P. operculella on Four Different Potato Tuber Varieties

Population parameters were calculated based on data from the entire cohort [20]. The intrinsic rate of increase (*r*), finite rate of increase (*λ*), net reproductive rate (*R*_0_), and mean generation time (*T*) of *P. operculella* on different tubers were calculated using the bootstrap method and listed in Table 2. Statistical analyses showed that the *r*, *λ*, *R*_0_, and *T* were negatively affected in *P. operculella* fed on Qingshu 9. For example, the *r*, *λ*, *R*_0_ fed on Lishu 6 were (*r* = 0.1434 per day, *λ* = 1.1542 per day, *R*_0_ = 67.6611 offspring per female) statistically significantly and higher than that of *P. operculella* fed on Yunshu 304 (*r* = 0.1180 per day, *λ* = 1.1253 per day, *R*_0_ = 39.5944 offspring per female), Hezuo 88 (*r* = 0.1156 per day, *λ* = 1.1226 per day, *R*_0_ = 35.2111 offspring per female), and Qingshu 9 (*r* = 0.0226 per day, *λ* = 1.0228 per day, *R*_0_ = 2.0556 offspring per female) (all *p*-values < 0.001). In addition, *T* was significantly shorter for *P. operculella* fed on Lishu 6 (29.3945 d) than for *P. operculella* fed on Hezuo 88 (30.7987 d), Yunshu 304 (31.1639 d), and Qingshu 9 (31.9294 d) (*p* < 0.01).

### 3.3. Pupal Weight of P. operculella on Four Different Potato Tuber Varieties

**Figure 3:**
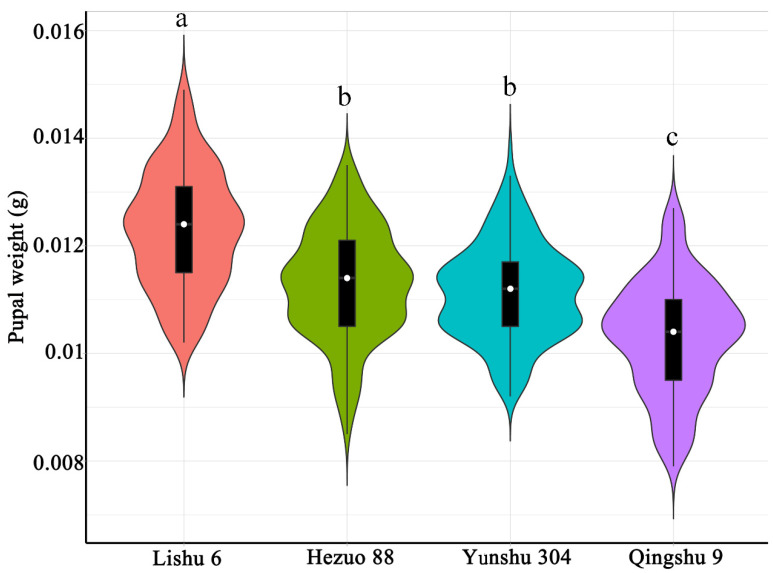
Pupal weight of *P. operculella* on four different potato tuber varieties. Different lowercase letters indicate means are significantly different at *p* < 0.001.

The weight of *P. operculella* differed among four different potato tuber varieties. The pupal weight on Lishu 6 (0.0123 g) was significantly heavier than that of *P. operculella* fed Hezuo 88 (0.0112 g), Yunshu 304 (0.0111 g), and Qingshu 9 (0.0103 g) (*F* = 132.4; df = 3; *p* < 0.001; Figure 3).

### 3.4. Oviposition Preference of P. operculella on Four Different Potato Tuber Varieties

**Figure 4:**
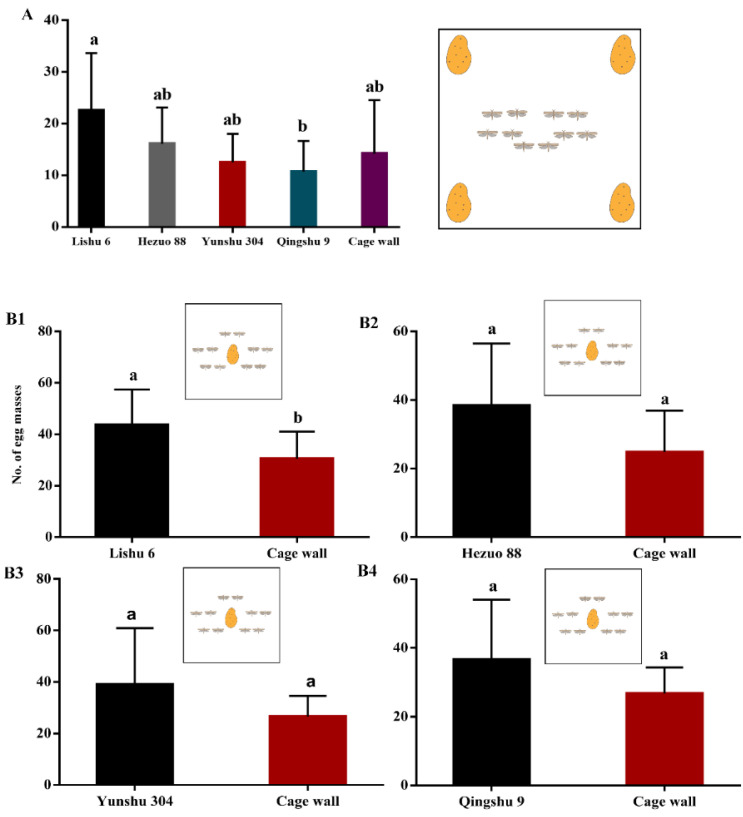
Number of egg masses laid by *P. operculella* on four different potato tuber varieties and the cage wall. Different lowercase letters indicate means are significantly different at *p* < 0.05. (**A**): choice test; (**B1**–**B4**): non-choice test.

Eggs occasionally are found on the surface of tubers, most commonly associated with indentations on the bud eyes, but they are usually not found inside tubers. In the choice test, *P. operculella* females preferred to oviposit on Lishu 6, followed by Hezuo 88, Yunshu 304, and then Qingshu 9 (*F* = 3.035; df = 4; *p* < 0.05; Figure 4A). In the non-choice test with individuals on four different potato tuber varieties, *P. operculella* females oviposited on each tuber variety (Figure 4B). In both choice and non-choice tests, *P. operculella* females also laid egg masses on the inner wall of the cage or the Petri dishes (Figure 4).

### 3.5. Feeding Preference of P. operculella Larvae on Four Different Potato Tuber Varieties

**Figure 5:**
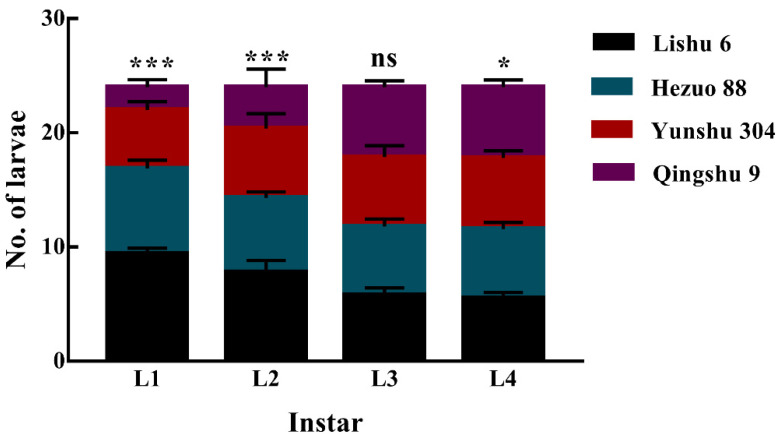
Number of *P. operculella* larvae fed on four different potato tuber varieties. Different lowercase letters indicate means are significantly different at ns, * *p* < 0.05; *** *p* < 0.001.

In free choice tests, the number of *P. operculella* 1st instar larvae on Lishu 6 (9), followed by Hezuo 88 (8), Yunshu 304 (5), and then Qingshu 9 (2) (*F* = 232.6; df = 3; *p* < 0.001; Figure 5). However, the capacity of larvae selected was decreased with the instar growth. There was no difference between the numbers of 3rd instar larvae among four different tuber varieties. However, *P. operculella* 4th instar larvae preferred to feed on Yunshu 304 (6.2), Qingshu 9 (6.2), and Hezuo 88 (6.1), but significantly higher on Lishu 6 (5.5) (*F* = 3.238; df = 3; *p* < 0.05).

## 4. Discussion

Foot [25] compared 20 potato cultivars for foliar and tuber resistance with negative results of *P. operculella*. The International Potato Center tested 3747 and 452 germplasms of primitive and wild potato species, respectively, from which 22 primitive and 21 wild germplasms were found resistant [26]. Malakar and Tingey [27] showed that some potato hybrids can inhibit oviposition, while surviving larvae had higher mortality and slower feeding rates than those larvae reared on commercial tubers. Rondon et al. [4] has determined tuber resistance of potato germplasm based on the number of mines per tuber and the number of live larvae. Therefore, a thorough knowledge of the biological performance of a pest among different varieties is necessary before developing management strategies, which can deliver a long-term management base to reduce the pest incidence in endemic areas [1]. The age-stage two-sex life table can furnish an inclusive and accurate description for insect populations performance under specific experimental conditions [28,29], which can consider an important index for screening varieties with high natural resistance. In this work, firstly, we examined the larval performance of *P. operculella* on four different tuber varieties (Lishu 6, Hezuo 88, Ynunshu 304, and Qingshu 9) using the age-stage two-sex life table method, which allowed us to compare the different performances among four potato tuber varieties and helped to screen natural resistance tubers. We determined significantly different survival and developmental times of lab-reared *P. operculella* through four host tubers. Compared with *P. operculella* larvae reared on the other three tubers, *P. operculella* larvae reared on Qingshu 9 showed the lowest survival rate and development, only 5.5% of the larval population developed into adults. Pupal weight also is an important factor of development of an insect’s reproductive system [30]. Although *P. operculella* was capable of successfully completing its whole life history on Qingshu 9, the weight of pupal individuals was smallest and the survival rate was lowest than those of *P. operculella* fed on the other three tuber varieties. The limited mobility of *P. operculella* larvae indicates that they often feed on the host plant preferred by their mothers [31]. Thus, female oviposition preference of *P. operculella* is extremely important for host selection [32]. The selection of suitable host tubers could be integral for *P. operculella* larvae in terms of their survival and development. Here, we compared female oviposition preference among four different tuber varieties. The results demonstrated that females oviposited on Lishu 6 in preference to Qingshu 9, consistent with the newly hatched larval preference on Lishu 6 than on Qingshu 9 tuber varieties, indicating that Qingshi 9 were of poorer quality as a food source for *P. operculella* larvae than the other three varieties. Moreover, we have found that larvae were willing to bore into tubers near eyebuds, and we knew from observations that adults preferred oviposit eggs eyebuds to other parts of tuber. Our findings put forward that *P. operculella* females select those hosts where larvae after hatching will flourish, compatible with the hypothesis of preference–performance [31,33]. The delayed developmental rate and minimum fertility of *P. operculella* on Qingshu 9 would yield to lowest populations resulting to lower pest infestation. These findings indicate that Lishu 6 is the optimal host, Hezuo 88 and Yunshu 304 are intermediate hosts, and Qingshu 9 is the least suitable host. The selected four varieties were most commonly planted and largely market demanding in Southwest China, especially Lishu 6 and Qingshu 9. Thus, it was necessary for Lishu 6 to use other pest management strategies, such as pesticides.

Malakar-Kuenen and Tingey [27] concluded that the thicker periderm thickness of tubers was responsible to slow down the penetration of larvae inside tubers and to initiate the successful establishment. Horgan et al. [34] reported a negative relationship between tuber cortex thickness and *P.* *operculella* pupal weight. Moreover, high levels of unidentified cortex-based defenses in tubers of *S. wittmackii* (Bitt.) could lead to a high proportion of larvae that were abandoned or died on the tubers (Horgan et al. [34]). Mansouri et al. [14] has indicated that the tuber flesh firmness negatively affect larval developmental time and female reproduction of *P. operculella*. In our research, although the flesh firmness of each variety was not studied, Qingshu 9 was thicker than the other three varieties according to the visual sense. The nutritional value of the host is an important resistance factor limiting normal growth and development of *P. operculella* [35]. Components of host plants, such as carbon, nitrogen, and defensive metabolites, directly affect insect fecundity [30]. Moreover, a previous study has demonstrated that there was a positive correlation between larval survival with the amounts of essential components (e.g., starch and minerals content) on potato. Learning the relationship between larval survival and nutrients or defensive metabolites can form a better understanding between host and *P. opercuella* damage.

Varietal selection can offer some opportunities to reduce *P. operculella* damage. Moreover, screening potato varieties with natural resistance is a direct strategy against the burrowing pest. Therefore, fully understanding the biological performance can provide basis for pest management. Our previous study also indicated that Lishu 6 plants are susceptible to *P. operculella*, and Qingshu 9 plants are not sensitive to *P. operculella* (unpublished data). Combining both results from plants and tubers studies, Qingshu 9 will be considered a good potato verity for IPM strategies for *P. operculella* in field conditions.

## 5. Conclusions

Our findings indicated that Lishu 6 is the optimal host, and Qingshu 9 is the least suitable host. This study provides the theoretical guidance for further management of *P. operculella* in the field.

## Figures and Tables

**Table 1:** Developmental time, longevity, and mean fecundity of *P. operculella* on four different potato tuber varieties.

Developmental Stage	Lishu 6		Hezuo 88		Yunshu 304		Qingshu 9	
	n	Developmental Time (d)	n	Developmental Time (d)	n	Developmental Time (d)	n	Developmental Time (d)
Egg	180	4.0 ± 0.0	180	4.0 ± 0.0	180	4.0 ± 0.0	180	4.0 ± 0.0
larval	180	14.18 ± 0.14 b	180	14.30 ± 0.11 b	180	14.37 ± 0.06 b	180	15.50 ± 0.34 a
Pupa	136	8.69 ± 0.09 c	106	9.07 ± 0.07 b	118	9.30 ± 0.09 a	14	9.90 ± 0.31 a
Female	58	14.28 ± 0.56 a	44	13.33 ± 2.30 a,b	50	10.74 ± 0.32 b	5	9.40 ± 1.05 b
Male	68	18.03 ± 0.27 a	54	14.72 ± 0.22 b	55	14.64 ± 0.24 b	5	12.80 ± 1.85 b
Mean fecundity/egg	58	213.67 ± 7.03 a	44	147.40 ± 5.71 b	50	142.54 ± 5.85 b	5	74.00 ± 6.26 c

Note: Values followed by the different lowercase letters within a row are significantly different using paired bootstrap test (*p* < 0.01).

**Table 2:** Population parameters of *P. operculella* on four different potato tuber varieties.

Parameter	Lishu 6	Hezuo 88	Yunshu 304	Qingshu 9
Intrinsic rate of increase, *r* (d^−1^)	0.1434 ± 0.0042 a	0.1156 ± 0.0046 b	0.1180 ± 0.0042 b	0.0226 ± 0.0159 c
Finite rate of increase, *λ* (d^−1^)	1.1542 ± 0.0048 a	1.1226 ± 0.0051 b	1.1253 ± 0.0047 b	1.0228 ± 0.0161 c
Net reproductive rate, *R*_0_ (offspring)	67.6611 ± 7.7254 a	35.2111 ± 4.8706 b	39.5944 ± 4.9943 b	2.0556 ± 0.9100 c
Mean generation time, *T* (d)	29.3945 ± 0.2516 c	30.7987 ± 0.2072 b	31.1639 ± 0.1834 a,b	31.9294 ± 1.1317 a

Note: Values followed by the different lowercase letters within a row are significantly different using paired bootstrap test (*p* < 0.01).
